# Urinary Concentrations of Triclosan in the U.S. Population: 2003–2004

**DOI:** 10.1289/ehp.10768

**Published:** 2007-12-07

**Authors:** Antonia M. Calafat, Xiaoyun Ye, Lee-Yang Wong, John A. Reidy, Larry L. Needham

**Affiliations:** Division of Laboratory Sciences, National Center for Environmental Health, Centers for Disease Control and Prevention, Atlanta, Georgia, USA

**Keywords:** 2,4,4′-trichloro-2′-hydroxydiphenyl ether, 5-chloro-2-(2,4-dichlorophenoxy)-phenol, biomonitoring, exposure, human, Irgasan, NHANES 2003–2004, urine

## Abstract

**Background:**

Triclosan is a synthetic chemical with broad antimicrobial activity that has been used extensively in consumer products, including personal care products, textiles, and plastic kitchenware.

**Objectives:**

This study was designed to assess exposure to triclosan in a representative sample ≥ 6 years of age of the U.S. general population from the 2003–2004 National Health and Nutrition Examination Survey (NHANES).

**Methods:**

We analyzed 2,517 urine samples using automated solid-phase extraction coupled to isotope dilution–high-performance liquid chromatography–tandem mass spectrometry.

**Results:**

We detected concentrations of total (free plus conjugated) triclosan in 74.6% of samples at concentrations of 2.4–3,790 μg/L. The geometric mean and 95th percentile concentrations were 13.0 μg/L (12.7 μg/g creatinine) and 459.0 μg/L (363.8 μg/g creatinine), respectively. We observed a curvilinear relation between age and adjusted least square geometric mean (LSGM) concentrations of triclosan. LSGM concentrations of triclosan were higher in people in the high household income than in people in low (*p* < 0.01) and medium (*p* = 0.04) income categories.

**Conclusions:**

In about three-quarters of urine samples analyzed as part of NHANES 2003–2004, we detected concentrations of triclosan. Concentrations differed by age and socioeconomic status but not by race/ethnicity and sex. Specifically, the concentrations of triclosan appeared to be highest during the third decade of life and among people with the highest household incomes.

Triclosan (2,4,4′-trichloro-2′-hydroxydiphenyl ether) is a synthetic, broad-spectrum antimicrobial agent that has been used extensively for more than 20 years in a variety of consumer products, including toothpaste, mouthwash, deodorants, soaps, textiles (e.g., socks, underwear), toys, liquid dishwashing soap, and plastic kitchenware (Adolfsson-Erici et al. 2002; Bhargava and Leonard 1996; Jones et al. 2000; National Library of Medicine 2007; Perencevich et al. 2001). In Europe, about 350 tons of triclosan are produced annually for commercial applications (Singer et al. 2002). In the United States, 76% of 395 commercial soaps examined contained triclosan (Perencevich et al. 2001). Despite its widespread use, the efficacy of triclosan-containing products in household and other non-health care–related settings and the potential hazards associated with this use, such as the emergence of antibiotic-resistant bacteria, are the subject of an ongoing scientific and public debate (Aiello et al. 2007; Jones et al. 2000; Kampf and Kramer 2004; Russell 2003; Weber and Rutala 2006; Yazdankhah et al. 2006).

Triclosan has been detected in the aquatic environment and in some food sources (Lindstrom et al. 2002; Lopez-Avila and Hites 1980; McAvoy et al. 2002; Okumura and Nishikawa 1996; Singer et al. 2002), and has attracted interest as an environmental contaminant (Halden and Paull 2005). In frogs, triclosan can disrupt thyroid hormone-associated gene expression and induce changes in the thyroid hormone-mediated metamorphosis process (Veldhoen et al. 2006). Triclosan can also alter circulating serum concentrations of total thyroxine in rats (Crofton et al. 2007). Triclosan is not acutely toxic to mammals (Bhargava and Leonard 1996), but it can interact with cytochrome P450–dependent enzymes, UDP-glucuronosyltransferases, and the human pregnane X receptor (Hanioka et al. 1996; Jacobs et al. 2005; Wang et al. 2004). The relevance of these interactions is unknown.

Information about the known commercial uses of triclosan indicates that ingestion and dermal absorption are the most likely routes of exposure (Moss et al. 2000; Sandborgh-Englund et al. 2006). Radioactive triclosan is excreted in feces, and to a lesser extent in urine, after topical exposure in rats (Moss et al. 2000) and oral administration in mice (Kanetoshi et al. 1988). The extent of dermal absorption of triclosan was examined both *in vitro* and *in vivo* in rats and humans (Moss et al. 2000). *In vitro*, 24 hr after application, about 6.3% of triclosan had penetrated human skin compared with 23% for rat skin. These data suggest that dermal absorption of triclosan in humans is lower than in rats. In all cases, glucuronidation and to a lesser extent sulfation of triclosan occurred during passage through the skin. No oxidative metabolites were detected in the urine *in vivo* or after absorption through skin *in vitro*; the major urinary metabolites were triclosan glucuronide and sulfate (Moss et al. 2000). In another study, 10 healthy adult Swedish volunteers (50% males; median age, 28 years) were exposed to a single oral dose of 4 mg triclosan by swallowing an oral mouthwash solution, and the volunteers’ plasma and urinary concentrations of triclosan were determined (Sandborgh-Englund et al. 2006). Triclosan concentrations in plasma increased rapidly, with a maximum concentration within 1–3 hr; the estimated terminal plasma half-life was 21 hr. The major fraction of the triclosan dose was excreted in urine within the first 24 hr (calculated urinary excretion half-life was 11 hr), and approached baseline levels within 8 days after exposure. A median oral dose of 54% was excreted in urine within the first 4 days after exposure. The percentage of free triclosan in plasma was higher than in urine; in urine, triclosan was excreted mostly in its conjugated forms. These data suggest that the concentrations of triclosan in urine (conjugated and free species) can be used as biomarkers of exposure to triclosan.

The widespread use of triclosan has raised interest about assessing human exposure to this compound. Therefore, we measured the urinary concentration of triclosan in participants of the National Health and Nutrition Examination Survey (NHANES) 2003–2004 to obtain the first nationally representative concentration of triclosan in the United States.

## Materials and Methods

NHANES, conducted annually since 1999 by the Centers for Disease Control and Prevention (CDC), is an ongoing survey designed to measure the health and nutritional status of the civilian noninstitutionalized U.S. population (CDC 2003). The surveys include household interviews, standardized physical examinations, and collection of medical histories and biologic specimens. Some of these specimens are used to assess exposure to environmental chemicals (CDC 2005). NHANES 2003–2004 included examinations of 9,643 people (CDC 2006a). Urine specimens for analyses of triclosan were collected from a random one-third subset of 2,517 participants ≥ 6 years of age. Because the subset was random, the representative design of the survey was maintained. The National Centers for Health Statistics Institutional Review Board reviewed and approved the study protocol. Informed written consent was obtained from all participants.

A single spot urine sample per participant was collected during one of three daily examination session periods (i.e., morning, afternoon, evening). The samples were shipped on dry ice to CDC’s National Center for Environmental Health and stored at or below −20°C until analyzed. We measured the concentrations of free plus conjugated triclosan, in 100 μL of urine, by online solid-phase extraction coupled to high-performance liquid chromatography–isotope dilution–tandem mass spectrometry, as described in detail elsewhere (Ye et al. 2005). Briefly, the conjugated species of triclosan were hydrolyzed by use of 50 μL of a solution (4,000 μg/mL) of β-glucuronidase/sulfatase (*Helix pomatia*, 463,000U/g solid; Sigma Chemical Co., St. Louis, MO) in 1 M ammonium acetate pH 5 buffer (Sigma Chemical Co.). After hydrolysis, samples were acidified with 0.1M formic acid; triclosan was preconcentrated by online solid-phase extraction, separated from other urine components by reversed-phase high-performance liquid chromatography, and detected by atmospheric pressure chemical ionization–tandem mass spectrometry. The limit of detection (LOD)—calculated as 3 *S*_0_, where *S*_0_ is the standard deviation as the concentration approaches zero (Taylor 1987)—was 2.3 μg/L; the precision ranged from 14.3% to 23.2%. To minimize potential contamination with triclosan during the laboratory operations, we avoided the use of triclosan-containing soaps and detergents. In addition, low-concentration (~ 40 μg/L) and high-concentration (~ 230 μg/L) quality control materials, prepared with pooled human urine, and reagent blanks (to monitor and control for the potential contamination arising from the reagents and apparatus used) were analyzed with analytical standards and NHANES samples.

We performed statistical analyses using SAS (version 9.1.3; SAS Institute Inc., Cary, NC) and SUDAAN (version 9.0.1; RTI International, Research Triangle Park, NC). SUDAAN incorporates sample weights and design variables to account for the complex sample design of NHANES. We calculated the percentage of detection, the geometric mean, and distribution percentiles for both the volume-based (in micrograms per liter) and creatinine-corrected (in micrograms per gram creatinine) concentrations. For concentrations below the LOD, as recommended for the analysis of NHANES data (CDC 2006b), we used a value equal to the LOD divided by the square root of 2 (Hornung and Reed 1990).

We used analysis of covariance to examine the influence of several variables, selected on the basis of statistical, demographic, and biologic considerations, on the concentrations of triclosan. For the multiple regression models, we used the variables described below and all possible two-way interactions to calculate the adjusted least square geometric mean (LSGM) concentrations (in micrograms per liter), which provide geometric mean estimates for a variable after adjustment for the model covariates. Because the distribution of the triclosan concentrations was skewed, triclosan concentrations were log-transformed. A variable based on self-reported data defined three major racial/ethnic groups: non-Hispanic black, non-Hispanic white, and Mexican American. Self-reported annual household income was available in $5,000 increments (ranging from < $5,000 to > $75,000). To obtain comparable numbers of participants in each income group, we categorized income as < $20,000, $20,000–$45,000, and > $45,000. Those participants who had serum cotinine concentrations (the biomarker used to define smoking status) > 10 μg/L were classified as smokers. Creatinine concentrations were log-transformed for the data analysis because of their skewed distribution. Age was reported in years at the previous birthday. Because body mass index (BMI) is age- and sex-specific for people < 19 years of age, CDC recommends for children and teens the use of BMI-for-age percentile (BMIPCT) instead of BMI (Kuczmarski et al. 2002). Therefore, we conducted two separate models: one for adults (≥ 20 years of age) and one for children and teenagers (6–19 years of age). We could not include only children (6–11 years of age) in the model because of the small sample size for some strata. We considered age (continuous), sex, race/ethnicity, creatinine concentration (Barr et al. 2005), and income for both models. Additionally, for the adult model, we included smoking status and BMI, and for the children and teens model, we included BMIPCT. When both age and age-squared were in the model, to avoid multicollinearity we centered age by subtracting the mean age from each participant’s age (Bradley and Srivastava 1979). To evaluate the relation between the log-transformed concentration of triclosan and age, we estimated the weighted geometric mean and LSGM concentrations after adjusting by the other covariates in the model, and we generated a bar chart of triclosan concentrations by age group.

To reach the final model, we used backward elimination, with a threshold of *p* < 0.05 for retaining the variable in the model, using Satterwaite-adjusted *F* statistics. We evaluated for potential confounding by adding each of the excluded variables back into the final model one by one and examining changes in the β coefficients of the statistically significant main effects. If addition of one of these excluded variables caused a change in a β coefficient by ≥ 10%, the variable was re-added to the model.

## Results

Free plus conjugated species of triclosan (total triclosan) were detected in 74.6% of the 2,517 urine samples from NHANES 2003–2004 at concentrations ranging from above 2.3 μg/L to 3,790 μg/L (2,644 μg/g creatinine). The geometric mean and 95th percentile concentrations were 13.0 μg/L (12.7 μg/g creatinine) and 459.0 μg/L (363.8 μg/g creatinine), respectively (Table 1).

The children and adolescents model included age (*p* = 0.4), age-squared (*p* = 0.04), income, log-transformed creatinine, race/ethnicity, and interaction terms between race/ethnicity and income (*p* = 0.04) and log-transformed creatinine and income (*p* = 0.02). However, the relatively low frequency of detection of triclosan (55%) in one of the combination groups (non-Hispanic whites with $20,000–$45,000 household income), although with sufficient sample size, resulted in a biased low LSGM and hence in the significant interaction term between race/ethnicity and income. Therefore, we repeated the multiple regression analyses without these interaction terms. The final model included income (*p* = 0.0014), log-transformed creatinine (*p* < 0.001), age (*p* = 0.12), and age-squared (*p* = 0.014) (Table 2). We observed both an accelerated increasing relationship between the log-transformed triclosan concentration and age (β coefficient for age-squared = 0.006), and a linear increasing relationship between the log-transformed triclosan and creatinine concentrations (β coefficient = 0.0002). People in the < $20,000 income group had lower LSGM [95% confidence interval (CI)] triclosan concentrations [9.3 μg/L (6.8–12.7 μg/L)] than those in the > $45,000 income group [15.7 μg/L (11.8–20.8 μg/L)]. People in the $20,000–$45,000 income group had the lowest LSGM concentrations [7.7 μg/L (5.6–10.8 μg/L)]. The frequency of detection of triclosan varied by income group [63.7% ($20,000–$45,000); 78.7% (< $20,000); and 76.8% (> $45,000)].

In the adult model, log-transformed creatinine, income, and age were significant (Table 2). Triclosan LSGM concentrations (95% CI) increased with household income and were significantly lower for people in the low household income category [10.2 μg/L (8.5–12.2 μg/L)] than for people in the medium [13.8 μg/L (10.9–17.4 μg/L); *p* = 0.02] and high [15.5 μg/L (13.7–17.5 μg/L); *p* = 0.01] income categories; the differences in LSGM concentrations between people in the medium and high household income categories were not statistically significant (*p* = 0.38). Triclosan concentrations decreased as age increased (β coefficient = −0.004) and increased as creatinine (log-transformed) increased (β coefficient = 0.58).

**Figure 1:**
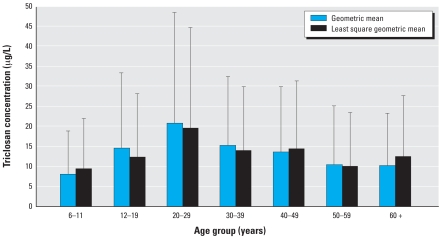
Geometric mean and least square geometric mean concentrations of triclosan (adjusted by income, age, and log of creatinine concentrations) by age. Error bars indicate 95% CIs.

Because BMI or BMIPCT and smoking status were not significantly associated with the triclosan concentration in the models above, we combined the two models for all ages without including these variables. In the final all-ages model, log-transformed creatinine, household income, age, and age-squared were significant (Table 2). The LSGM urinary triclosan concentration (in micrograms per liter) increased as income increased: Participants in the high household income category had significantly higher LSGM (95% CI) triclosan concentrations [15.3 (13.7–17.3)] than participants in the low-[10.3 (9.1–11.6); *p* < 0.01] and medium-[12.2 (10–14.9); *p* = 0.04] income categories. However, differences in LSGM concentrations between people in the medium and lowest household income categories were not statistically significant (*p* = 0.13). The triclosan concentrations increased as creatinine (log-transformed) increased (β coefficient = 0.63). Figure 1 shows the relationship of triclosan concentrations with age.

The univariate analyses showed that regardless of the examination session time, the geometric mean triclosan concentrations were not significantly different (all *p-*values > 0.25). Furthermore, the final multiple regression model did not include examination session time (*p* = 0.22). These results suggest that the time of collection of the sample was not associated with the urinary concentration of triclosan.

## Discussion

We detected concentrations of free plus conjugated species of triclosan in urine in 74.6% of the samples examined. This high frequency of detection is most likely associated with daily use by the U.S. general population of consumer products that contain triclosan, including at least one toothpaste brand (Food and Drug Administration 1997), skin-care products (e.g., soap, deodorant, skin cleanser), and other household products (e.g., pet care, cleaners) (National Library of Medicine 2007). In humans, triclosan can be absorbed through skin (Moss et al. 2000) and through the mucosa in the mouth and intestinal tract (Lin 2000; Sandborgh-Englund et al. 2006). The detection of triclosan in blood (Hovander et al. 2002; Sandborgh-Englund et al. 2006), urine (Sandborgh-Englund et al. 2006; Wolff et al. 2007; Ye et al. 2005), and milk (Allmyr et al. 2006) collected from small groups of persons in the United States and Sweden suggests that the general population is exposed to triclosan.

The range of urinary concentrations of triclosan in the NHANES 2003–2004 sample was wide, with 25% of persons examined having concentrations < 2.3 μg/L, and 5% of the participants having concentrations > 363.8 μg/g creatinine (Table 1). A wide distribution of concentrations of triclosan has also been reported for 10 healthy Swedish volunteers, five of whom related using personal-hygiene products that contained triclosan (Sandborgh-Englund et al. 2006). In the Swedish study, the baseline urinary excretion of triclosan (determined from 24-hr urine samples) was 0.1–743 μg/day among people not using triclosan-containing products, and 21–218 μg/day among users of triclosan-containing products. In another Swedish study involving a group of 36 nursing women, triclosan concentrations were higher in both plasma and milk among the women who used personal care products containing triclosan than in the women who did not (Allmyr et al. 2006). These data suggest that personal care products may be a principal source of exposure to triclosan in humans. The wide range of concentrations of triclosan may be attributable to differences in exposure, as well as to individual variations in distribution kinetics and metabolism (Sandborgh-Englund et al. 2006).

Data are limited on the urinary concentrations of triclosan in human populations. In a pilot study, triclosan was detected in 67.8% of 90 prepubertal girls, with mean age of 7.77 years, from New York City, New York; Cincinnati, Ohio; and Northern California (Wolff et al. 2007). The median concentration (5.9 μg/L) was comparable to the median concentration of triclosan for the 341 children 6–11 years of age in this NHANES 2003–2004 population (7.2 μg/L).

As is true for other nonpersistent chemicals (Fenske et al. 2005; Hauser et al. 2004; Hoppin et al. 2002; Meeker et al. 2005), within-person variability in urinary concentrations of triclosan exists. Despite this variability, results from one recent study suggest that triclosan concentrations in a single urine sample can be used to categorize the 6-month average exposure to triclosan among a group of 35 children (Teitelbaum et al. 2007). More important, concentrations based on one spot sample per person can be useful in calculating mean population concentration estimates in cross-sectional studies such as NHANES.

We observed a curvilinear-increased relation between age and triclosan LSGM concentration for people ≥ 6 years of age. For people ≥ 20 years of age, concentrations appeared to decline as age increases (Figure 1 and Table 2). These data suggest that the concentrations of urinary species of triclosan peak around the third decade of life and then slowly decrease. This relation between age and triclosan concentration is not clearly understood, and these differences might reflect differences in lifestyle choices affecting exposure and/or pharmacokinetic factors based on age.

We did not observe differences in the adjusted LSGM concentrations of triclosan based on race/ethnicity or sex. LSGM triclosan concentrations were significantly higher among people in the high household income category than among people in the medium (*p* = 0.04) and low (*p* < 0.01) income categories. These differences might reflect differences in lifestyle choices (e.g., use of personal care products) that affect exposure to triclosan.

In summary, these NHANES 2003–2004 triclosan data can be used to establish a nationally representative baseline assessment of exposure, a baseline to which the triclosan concentrations in future populations can be compared to identify exposure trends. The reported high frequency of detection of triclosan and the differences in urinary concentrations based on age and socioeconomic status highlight the importance of additional research to identify the sources and potential routes of human exposure to triclosan. In addition, these data provide exposure information that can be useful for risk assessment if toxicologic or epidemiologic studies so indicate.

## Figures and Tables

**Table 1:** Geometric mean and selected percentiles of triclosan concentrations [μg/L (95% CI)] in urine for the U.S. population ≥ 6 years of age: data from NHANES 2003–2004.

Variable^a^	Geometric mean	10th percentile	25th percentile	50th percentile	75th percentile	90th percentile	95th percentile	No.
All	13.0 (11.6–14.6)	< LOD	< LOD	9.2 (7.9–10.9)	47.0 (37.9–58.4)	249.0 (188.0–304.0)	459.0 (386.0–522.0)	2,517
	12.7 (11.5–14.1)	< LOD	< LOD	9.5 (8.2–10.4)	43.8 (33.8–60.2)	212.1 (174.0–251.0)	363.8 (294.4–462.8)	2,514
6–11 years	8.2 (6.2–10.8)	< LOD	< LOD	5.9 (4.0–8.5)	20.5 (14.3–31.6)	123.0 (36.4–163.0)	148.0 (110.0–273.0)	314
	9.9 (7.4–13.3)	< LOD	< LOD	7.5 (4.7–13.4)	24.1 (15.3–35.6)	115.3 (39.9–235.6)	226.4 (115.3–336.3)	314
12–19 years	14.5 (11.0–19.1)	< LOD	2.8 (< LOD–4.0)	10.2 (8.2–13.1)	39.0 (26.5–86.4)	304.0 (134.0–566.0)	649.0 (310.0–890.0)	715
	10.9 (8.3–14.2)	< LOD	2.9 (< LOD–3.7)	7.4 (5.5–10.7)	31.8 (21.9–61.1)	193.1 (90.7–317.9)	347.2 (169.4–579.9)	713
20–59 years	14.7 (13.1–16.5)	< LOD	2.7 (< LOD–3.6)	10.3 (8.8–12.5)	57.0 (41.5–73.2)	264.0 (208.0–352.0)	491.0 (418.0–554.0)	951
	13.8 (12.4–15.3)	< LOD	3.2 (2.7–3.6)	10.6 (9.1–11.7)	53.3 (39.1–77.6)	224.3 (175.3–272.2)	384.5 (294.4–500.0)	950
≥ 60 years	10.3 (8.0–13.1)	< LOD	< LOD	6.5 (3.9–11.2)	41.1 (20.9–60.9)	197.0 (142.0–270.0)	386.0 (299.0–470.0)	537
	12.4 (9.7–15.9)	< LOD	< LOD	8.5 (6.9–10.6)	39.8 (21.4–93.9)	216.7 (157.5–307.8)	382.8 (278.8–700.0)	537
Female	10.6 (9.3–12.1)	< LOD	< LOD	7.4 (6.1–9.1)	33.2 (27.1–39.4)	144.0 (96.5–250.0)	363.0 (258.0–430.0)	1,288
	12.3 (10.6–14.2)	< LOD	< LOD	9.5 (8.4–10.4)	32.3 (26.2–46.6)	181.8 (138.3–216.7)	331.5 (225.0–479.6)	1,286
Male	16.2 (13.4–19.6)	< LOD	2.7 (< LOD–3.8)	11.7 (9.3–14.8)	83.3 (50.6–111.0)	310.0 (231.0–433.0)	566.0 (461.0–716.0)	1,229
	13.3 (11.3–15.6)	< LOD	2.7 (< LOD–3.4)	9.2 (6.9–12.1)	72.5 (45.8–85.9)	237.2 (175.3–294.4)	384.5 (294.4–506.0)	1,228
Mexican American	14.6 (10.6–20.1)	< LOD	< LOD	8.7 (5.3–17.5)	65.4 (32.8–127.0)	354.0 (225.0–456.0)	597.0 (372.0–992.0)	613
	13.3 (9.4–18.8)	< LOD	< LOD	9.2 (5.5–13.9)	66.6 (28.8–112.3)	291.6 (150.6–432.3)	446.0 (262.7–1147.2)	612
Non-Hispanic black	14.4 (11.4–18.2)	< LOD	3.6 (2.5–5.2)	11.1 (8.7–16.1)	37.3 (30.2–58.0)	203.0 (87.5–341.0)	446.0 (254.0–750.0)	652
	9.9 (7.9–12.5)	< LOD	< LOD	7.7 (5.5–10.0)	29.8 (25.5–37.3)	131.4 (78.0–212.9)	257.1 (126.9–513.4)	651
Non-Hispanic white	12.9 (11.2–14.9)	< LOD	< LOD	9.1 (7.4–11.0)	49.2 (37.8–63.4)	245.0 (163.0–334.0)	461.0 (383.0–527.0)	1,092
	13.3 (11.6–15.1)	< LOD	< LOD	9.8 (8.1–11.5)	47.0 (34.3–67.6)	212.9 (159.8–272.2)	356.4 (276.0–479.6)	1,091

CI, confidence interval. Blue lines denote measure in μg/g creatinine.

a Participants not defined by the three racial/ethnic groups shown were included only in the total population estimate. LOD = 2.3 μg/L.

**Table 2:** β-coefficients (*p*-values) for the significant variables from the multiple regression models of the triclosan urinary concentration (log-transformed).

Variable	Children and adolescents (6–19 years of age)	Adult (≥ 20 years of age)	All ages
Intercept	−0.51 (0.036)	0.21 (0.18)	−0.04 (0.73)
Creatinine concentration (log-transformed)	0.7903 (< 0.001)	0.58 (< 0.001)	0.63 (< 0.001)
Household income			
< $20,000	−0.23 (0.29)	−0.18 (0.005)	−0.18 (< 0.001)
$20,000–$45,000	−0.31 (0.82)	−0.05 (0.38)	−0.1 (0.04)
> $45,000	Reference	Reference	Reference
Age	0.0157 (0.12)	−0.004 (0.003)	0.0002 (0.86)
Age-squared	0.006 (0.01)		−0.0001 (< 0.001)
